# Azide-Alkyne Huisgen [3+2] Cycloaddition Using CuO Nanoparticles

**DOI:** 10.3390/molecules171113235

**Published:** 2012-11-06

**Authors:** Hyunje Woo, Hyuntae Kang, Aram Kim, Seongwan Jang, Ji Chan Park, Sungkyun Park, Byeong-Su Kim, Hyunjoon Song, Kang Hyun Park

**Affiliations:** 1 Department of Chemistry, Chemistry Institute for Functional Materials, Pusan National University, Busan 609-735, Korea; 2 Clean Fuel Department, Korea Institute of Energy Research, Daejeon 305-343, Korea; 3 Department of Physics, Pusan National University, Busan 609-735, Korea; 4 Interdisciplinary School of Green Energy and School of NanoBioscience and Chemical Engineering, Ulsan National Institute of Science and Technology (UNIST), 100 Banyeon-ri, Ulsan 689-798, Korea; 5 Department of Chemistry, Korea Advanced Institute of Science and Technology, Daejeon 305-701, Korea

**Keywords:** copper oxide, nanocatalyst, heterogeneous, hybrid, click reaction

## Abstract

Recent developments in the synthesis of SpellECuO nanoparticles (NPs) and their application to the [3+2] cycloaddition of azides with terminal alkynes are reviewed. With respect to the importance of click chemistry, CuO hollow NPs, CuO hollow NPs on acetylene black, water-soluble double-hydrophilic block copolymer (DHBC) nanoreactors and ZnO–CuO hybrid NPs were synthesized. Non-conventional energy sources such as microwaves and ultrasound were also applied to these click reactions, and good catalytic activity with high regioselectivity was observed. CuO hollow NPs on acetylene black can be recycled nine times without any loss of activity, and water-soluble DHBC nanoreactors have been developed for an environmentally friendly process.

## 1. Introduction

**Scheme 1:**
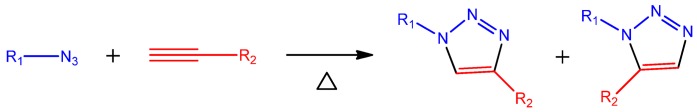
Azide-alkyne [3+2] cycloaddition generating 1,4 and 1,5 regioisomer.

Recently, metal oxide nanoparticles (NPs) have been used frequently as metal catalysts due to their high physical and chemical stability [1,2,3,4]. Their distinct qualities, particularly their large surface area, makes them applicable to a wide range of fields. Among the metal oxide NPs, copper oxides (Cu_2_O, CuO) are p-type semiconductor materials with a small band gap energy. Recently, Tarascon’s group used copper oxide (Cu_2_O, CuO) NPs as an anode material for lithium ion cells [5], while Izaki’s group employed n-type semiconducting material ZnO NPs, for solar cell plates, thereby demonstrating their highly useful electrochemical characteristics [6]. In addition, copper oxide (Cu_2_O, CuO) NPs have sufficient space to adsorb harmful gases, as shown by their application as a gas sensor by Yadong’s group [7]. Furthermore, Cu(II) NPs are non-toxic, environmentally friendly, highly stable, and recyclable. The research results presented here demonstrate the use of Cu(II) NPs for click chemistry. Click chemistry is a highly efficient cycloaddition method that became very popular as a new synthesis route since its introduction in 2001 by Sharpless [8]. This click chemistry concept as a wide scope, and it can be defined by the following criteria, high yield, modularity, readily available starting materials and reagents, and simple reaction conditions. The azide-alkyne Huisgen method, involving Cu(I)-catalyzed cycloaddition between terminal acetylenes and azides at room temperature is one of the most efficient reactions within the concept of click chemistry [9,10,11]. The reaction proceeds in variable solvents in the presence of a catalyst and yields stable triazoles with possible applications in pharmaceuticals, DNA modification, and organic synthesis [12]. The commonly used catalysts in click reaction are Cu and Ru. The Cu catalyst generates the 1,4-regioisomer, while Ru-based catalysis yields the 1,5-regioisomer (Scheme 1).

**Scheme 2:**
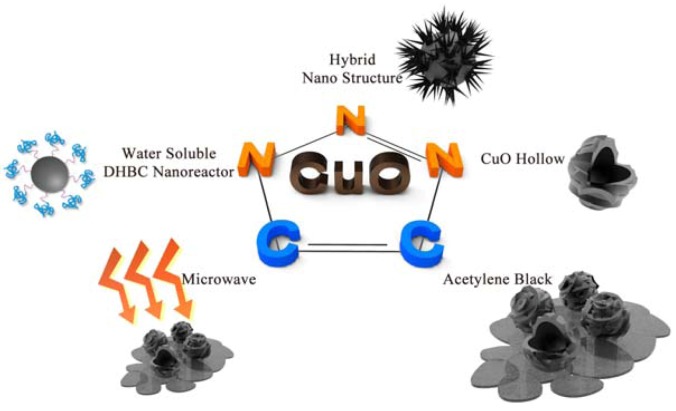
[3+2] Cycloaddition of azides with terminal alkynes using various CuO nanocatalysts.

Recently, CuO NPs have been already used for the preparation of nucleosides in biochemistry and as a catalyst in azide-alkyne cycloadditions by other groups [13,14]. Other groups have also synthesized CuO NPs as a catalyst for CSe, CTe, and CS bond formation and synthesis of 2-aminobenzothiazoles [15,16]. To increase the surface area for enhancing the catalytic activity, CuO hollow NPs have been synthesized by using Cu_2_O nanocube colloidal solutions [17]. Due to the large surface area of the hollow nanostructures, this material was applied in click chemistry as a highly efficient catalyst. Previously, it was not possible to recover and recycle homogeneous catalysts which posed the greatest problem in the pharmaceutical field. In the present study, CuO hollow NPs were anchored onto acetylene black (AB) in order to resolve the catalyst recovery problem [18]. In addition, the excitation of the reacting reagents by non-conventional energy sources such as microwaves and ultrasound represents well-known valuable techniques in organic chemistry. The use of these energy sources results in shorter reaction times compared to conventional heating [19]. Since the use of environmentally friendly substances has obvious ecological and economic advantages, performing click chemistry in aqueous media is becoming popular, but remains challenging [20,21,22,23,24,25]. Double-hydrophilic block copolymers (DHBCs), which consist of one active ionizable and one neutral block were used for the growth control of the inorganic phase. CuO NPs synthesized by using the DHBCs method, as well as hybrid nanostructures, were applied for ultrasound-assisted click chemistry [26]. The hybrid nanostructures exhibited the highest catalytic activity among conventional heterogeneous catalysts in ultrasound-assisted click chemistry [27] (Scheme 2).

## 2. Representative Experimental Methods

### 2.1. Preparation of Cu_2_O Nanocubes

Poly(vinylpyrrolidone) (PVP, Aldrich, Mw 55,000; 5.3 g) dissolved in 1,5-pentanediol (PD, Aldrich, 96%, 45 mL) was heated to 240 °C under inert conditions. Then, Cu(acac)_2_ (STREM, 98%, 4.0 mmol) dissolved in PD (15 mL) was injected into the hot PVP solution at 240 °C, and the mixture was stirred for 15 min at the same temperature. The colloidal dispersion was cooled to room temperature, and the product was separated by adding acetone (150 mL) with centrifugation at 8,000 rpm for 20 min. The precipitates were washed with ethanol several times, and re-dispersed in ethanol (50 mL).

### 2.2. Synthesis of CuO Hollow and Branched Nanostructures

An appropriate concentration of aqueous ammonia solution was added into the Cu_2_O cube dispersion in ethanol (25 mL, 16 mM with respect to the precursor concentration). The mixture was subjected to stirring at room temperature for 2 h. The volume and concentration of the aqueous ammonia solution used for each structure were 1.0 mL and 14.7 M for hollow cubes, 2.0 mL and 7.36 M for hollow spheres, and 6.0 mL and 2.45 M for urchin-like particles, respectively. For shape optimization of the hollow spheres, a 3.68 M aqueous ammonia solution was used. After the reaction, the products were collected by centrifugation at 6,000 rpm for 20 min. For the mechanism study, the appropriate amount of NaOH (Aldrich, 99.998%) was dissolved in aqueous ammonia solution (14.7 M, 1.0 mL). The resulting mixture was added to the ethanol dispersion of Cu_2_O cubes (25 mL, 16 mM with respect to the precursor concentration), and allowed to stir at room temperature for 2 h. Hollow cubes were obtained without NaOH. Addition of NaOH (20 mg, 0.50 mmol and 50 mg, 1.3 mmol) yielded hollow spheres and urchin-like particles, respectively.

### 2.3. Immobilization of CuO Hollow Nanospheres on Acetylene Carbon Black (CuO/AB) and Charcoal (CuO/C)

Acetylene carbon black (STREM, 99.99%, 1.2 g) was mixed with CuO hollow nanosphere dispersion in ethanol (17.0 mM, 100 mL), and the reaction mixture sonicated for 1 h at room temperature. After 1 h, the product CuO/AB was washed with ethanol several times and vacuum dried at room temperature. For the synthesis of CuO/C, a mixed solution of charcoal (0.8 g) and CuO hollow nanosphere dispersion in ethanol (50.0 mM, 50.0 mL) was refluxed for 4 h. After 4 h, the black suspension was cooled to room temperature and precipitated by centrifugation. The product CuO/C was washed with ethanol thoroughly and dried in a vacuum oven at room temperature.

### 2.4. Water-Soluble CuO NPs

The CuO NPs were prepared according to the following protocols: first, double-hydrophilic block copolymer PEO(3500)-b-PAA(7500) (Polymer Source Inc, Montereal, Canada, 21.15 mg, 0.20 mmol carboxylic-acid groups) and copper chloride dihydrate (17.8 mg, 0.10 mmol) in water (5.0 mL) were separately prepared and mixed under vigorous stirring. NaOH (1.0 M, 0.10 mL, 0.10 mmol) was added to this solution mixture, and a blue precipitate was obtained. Subsequently, 10.0 M hydrazine (0.10 mL, 1.0 mmol) was added dropwise to the resulting suspensions under vigorous stirring. As soon as hydrazine was added, the solution turned orange. After 20 min of vigorous stirring, the reaction mixture kept still for 10 min, after which the solution color changed to brownish red. The solution mixture was centrifuged to remove the large aggregates of particles therein (1,500 rpm, 15 min), then the recovered supernatant was used as a catalyst for click chemistry.

### 2.5. Synthesis of Polycrystalline ZnO Nanospheres

A mixture of zinc(II) acetylacetonate hydrate (0.10 g, 0.40 mmol) and PVP (1.0 g, 9.0 mmol) was dissolved in PD (40 mL), and then slowly heated to 230 °C for 12 min under an inert condition. The mixture solution was allowed to stir at the same temperature for 3 min. After that, the colloidal dispersion was cooled to room temperature, and the product was separated by adding ethanol (120 mL) with centrifugation. The precipitates were washed with ethanol several times and re-dispersed in ethanol (10 mL).

### 2.6. Synthesis of ZnO/Cu_2_O Hybrid Nanoparticles

A mixture of zinc(II) acetylacetonate hydrate (0.10 g, 0.40 mmol) and PVP (1.0 g, 9.0 mmol) was dissolved in PD (40 mL), and then slowly heated to 230 °C for 12 min under an inert atmosphere. The mixture was allowed to stir at the same temperature for 3 min. Then, Cu(acac)_2_ (0.1 g, 0.40 mmol) dissolved in PD (5.0 mL) was injected into the hot zinc-PVP mixture solution at 230 °C and the mixture was stirred for 10 min at the same temperature. The colloidal dispersion was cooled to room temperature, and the product was separated by adding ethanol (120 mL) with centrifugation. The precipitates were washed with ethanol several times and re-dispersed in ethanol (10 mL).

### 2.7. Synthesis of ZnO/CuO Core-Branch Nanoparticles

An aqueous sodium hydroxide solution (1.0 M, 1.0 mL) was added into ZnO/Cu_2_O hybrid nanoparticle dispersion in ethanol (25 mL, 8.0 mM with respect to the precursor concentration). The mixture was subjected to stirring at room temperature for 1 h. After the reaction, the product was collected by centrifugation. Finally, the particles were dispersed in ethanol.

## 3. CuO Hollow Nanoparticles: [3+2] Cycloaddition of Azides with Terminal Alkynes

**Figure 1:**
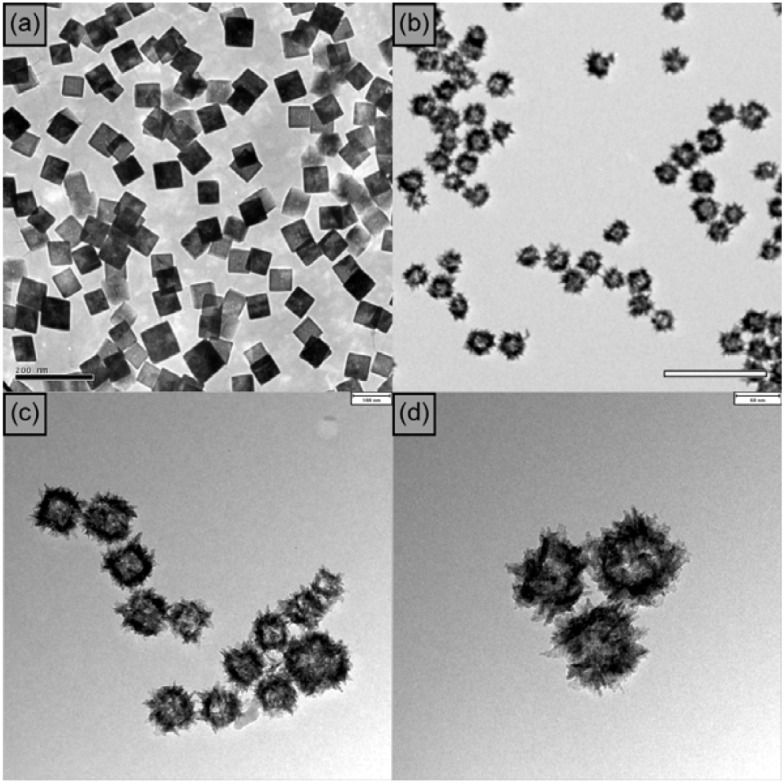
TEM images of (**a**) Cu_2_O nanocube; (**b**) CuO hollow nanoparticles; (**c**) CuO hollow nanoparticles after click reaction; and (**d**) magnification of CuO hollow nanoparticles. The scale bars represent 200 nm, 200 nm, 100 nm, 50 nm, respectively (**a**–**d**).

In general, a Cu(I) salt is directly used as a catalyst. Alternatively, Cu(II) may be used after reduction [28,29,30]. This study shows the best results of click reactions using well-designed, uniform, hollow-structured CuO nanoparticles (Figure 1). An approach for the gram-scale synthesis of uniform Cu_2_O nanocubes by a one-pot polyol process was used [31]. CuO hollow NPs were prepared by adding aqueous ammonia solutions to Cu_2_O nanocube colloidal solutions.

As shown in Table 1, several experiments were conducted in order to determine the solvent system most suitable for the catalyst. Considering the hygroscopic properties of THF, dioxane and toluene, it is expected that the reaction conditions will be affected by the amount of water present (entries 1–4, Table 1). Both *t*-BuOH [32] and water as solvents gave high yields under mild conditions (entries 5 and 6), though the best results were obtained with a solvent mixture of *t*-BuOH and H_2_O (2:1) (entry 4), which indicates that solubility and hygroscopic properties are comparably important factors. In case of using commercially available CuO, a yield of less than 1% was found under the same conditions. Extending the reaction time to 24 h, a yield of merely 35% was achieved (entries 7 and 8). Please note that even under optimum reaction conditions, no reaction occurred without catalyst (entries 9, Table 1).

**Table 1:** Optimization of click reaction catalyzed by various CuO(II) nanoparticles.

Entry	Cat (5 mol%)	Temp (°C)	Time (h)	Solvent	Conv. ^a^ (%)
1	CuO urchins	60	12	THF-H_2_O (24:1)	4
2	CuO urchins	100	12	Dioxane/H_2_O (24:1)	61
3	CuO urchins	110	12	Toluene/H_2_O (24:1)	93
4	CuO urchins	25	3	H_2_O/t-BuOH (2:1)	96
5	CuO urchins	25	3	H_2_O	90
6	CuO urchins	25	3	*t*-BuOH	71
7	Commercial CuO ^b^	25	24	H_2_O/*t*-BuOH (2:1)	35
8	Commercial CuO ^b^	25	3	H_2_O/*t*-BuOH (2:1)	<1
9	─	25	3	H_2_O/*t*-BuOH (2:1)	0
10	CuO urchins	25	3	H_2_O/*t*-BuOH (2:1)	93 ^c^
11	CuO hollow spheres	25	3	H_2_O/*t*-BuOH (2:1)	100 ^c^
12	CuO hollow cubes	25	3	H_2_O/*t*-BuOH (2:1)	94 ^c^
13 ^d^	CuO hollow spheres	25	0.5	H_2_O/*t*-BuOH (2:1)	98

*Reaction Conditions*: benzyl azide (1mmol) and phenylacetylene (1.5 mmol) in H_2_O-*t*-BuOH (2 : 1). ^a^ Determined by ^1^H-NMR spectra; ^b^ Purchased from Aldrich (nanopowder, cat No. 544868); ^c^ Conversion based upon an average of two runs; ^d^ In the presence of 1.0 eq. Et_3_N.

Generally, decreasing size of the particles and increasing active surface area lead to enhanced catalytic activity. However, CuO hollow spheres showed a slightly better activity than hollow cubes, as shown in Table 1. The corresponding Brunauer–Emmett–Teller (BET) surface areas of the different CuO NPs measured by nitrogen sorption experiments are 79 m^2^g^−1^ for hollow cubes, 113 m^2^g^−1^ for hollowspheres, and 81 m^2^g^−1^ for urchin-like particles. It is quite reasonable that the catalytic activities depend on the active surface areas of the catalysts. When Et_3_N (1.0 eq.) was added, the reaction proceeded faster and was completed within 30 min (entry 13, Table 1). After cycloaddition, the CuO hollow nanospheres were separated by centrifugation and subsequently used in click reactions with phenylacetylene at least three times without loss in catalytic activity. 

## 4. Immobilized CuO Hollow Nanospheres in Alkyne-Azide Cycloadditions

The impossibility in recovering and recycling homogeneous catalysts is a task of great economic and environmental importance in the chemical and pharmaceutical industries, especially when expensive and/or toxic heavy metal complexes are employed [33]. The development of catalysts anchored to solid supports has been one of the areas of most intense research activity over the past years. Acetylene black is a special type of carbon black formed by an exothermic decomposition of acetylene and is characterized by the highest degree of aggregation and crystalline orientation when compared with all types of carbon black. Acetylene black is widely used in battery systems possessing excellent electric conductivity, large specific surface areas and strong adsorptive abilities, as well as in supports [34]. 

**Figure 2:**
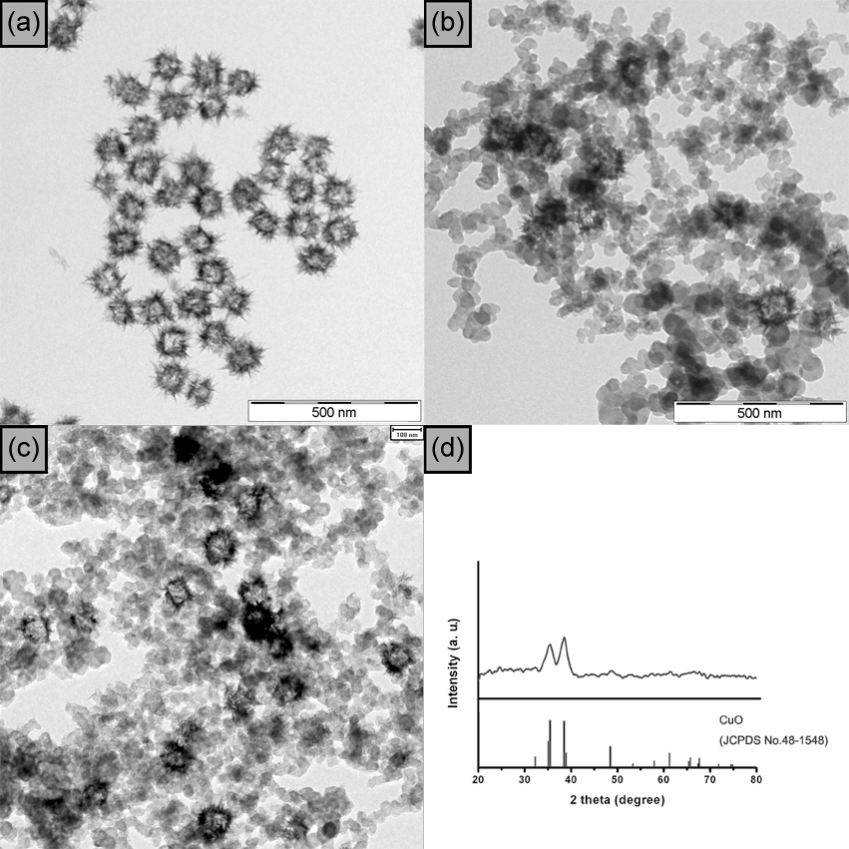
TEM images of (**a**) CuO hollow nanoparticles; (**b**) CuO hollow nanoparticles on acetylene black; and after fifth cycle (**c**); (**d**) XRD spectra of CuO hollow nanoparticles on acetylene black. The scale bars represent 500 nm, 500 nm, 100 nm, respectively (**a**–**c**).

The transmission electron microscopy (TEM) image in Figure 2a shows the regular shape of the CuO particles. CuO hollow spheres were obtained as highly monodisperse NPs with a size of 103 ± 8 nm. The crystalline features of the hollow spheres are represented in the XRD data (Figure 2d). The CuO hollow particles were immobilized on acetylene carbon black by simple sonication at room temperature. The TEM image in Figure 2b shows that the immobilized CuO hollow spheres are well dispersed and isolated with approximately 100 nm average diameter, thus maintaining their original size and structure. As shown in Figure 2c, the structure of the CuO hollow NPs onto AB remained unchanged after the reaction, thereby demonstrating the recyclability of the catalyst [17]. 

As shown in Table 2, the reaction was carried out at 25–50 °C using benzyl azide and phenylacetylene as the benchmark substrate. With respect to temperature and for *t*-BuOH being the solvent, the best results were obtained at mild, room temperature conditions. The results could be further improved by replacing the pure solvents *t*-BuOH and H_2_O by a corresponding mixture in the ratio 2:1, indicating that both solubility and hydroscopic properties are important factors. 

Remarkably, after the reaction, the CuO NPs on AB were separated by centrifugation and could be reused up to nine times under the same reaction conditions without any loss of catalytic activity. An inductively coupled plasma-mass spectrometry (ICP-AES) study showed that the copper loss from the catalyst was negligible. These results confirm that the catalytic system presented here satisfies the conditions for heterogeneous catalysts with respect to easy separation, recyclability, and persistence. Moreover, these heterogeneous systems are promising industrial catalysts.

**Table 2:** Optimization of click reaction catalyzed by various CuO nanoparticles.

Entry	Cat (mol%)	Temp (°C)	Time (h)	Conv (%) ^a^
1	Blank	50	5	7
2	CuO (5 mol%)	25	3	100
3	CuO on AB (1 mol%)	25	3	>1
4	CuO on AB (1 mol%)	50	5	22
5	CuO on AB (3 mol%)	25	5	>1
6	CuO on AB (3 mol%)	50	5	100
7	CuO on AB (3 mol%)	50	3	60
8	CuO on AB (3 mol%)	40	5	23
9	CuO on AB (3 mol%)	30	5	1.1
10	CuO on AB (5 mol%)	50	5	96
11	Recovered from # 6	50	5	100
12	Recovered from # 12	50	5	100
13	Recovered from # 13	50	5	100
14	Recovered from # 14	50	5	100
15	Recovered from # 15	50	5	100
16	Recovered from # 16	50	5	98
17	Recovered from # 17	50	5	100
18	Recovered from # 18	50	5	100
19	Recovered from # 19	50	5	100

*Reaction conditions*: benzyl azide (0.84 mmol) and phenylacetylene (1.18 mmol) in H_2_O-*t*-BuOH (2 : 1). ^a^ Determined by ^1^H-NMR. Yields are based on the amount of benzyl azide used.

## 5. Solvent-Free Microwave Promoted [3+2] Cycloaddition of Alkyne-Azide

**Scheme 3:**
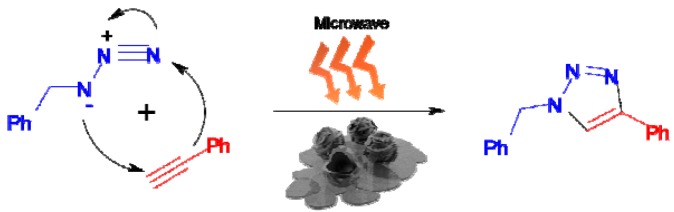
Microwave-assisted solvent-free click reaction.

Microwave-assisted organic synthesis (MAOS) has been highlighted as a very synthesis route due to the short reaction times in comparison to methods employing conventional heating [35,36,37]. Microwave activation with it non-conventional energy source is becoming a very popular and valuable technique in organic chemistry, as demonstrated by the annual publications on this topic, whose number is rapidly increasing. Thus, MAOS was employed for the chemical reactions presented herein (Scheme 3). 

As shown in Table 3, several experiments were conducted in order to determine the solvent system that is most suitable for the catalyst. The H_2_O/*t*-BuOH solvent system was employed to compare the synthesis by microwave activation and conventional heating. To complete the reaction by conventional heating, a reaction time of 5 h at 50 °C was required. When the temperature was increased to 100 °C, a 91% conversion yield was obtained in 1 h. The reaction time was shortened to 1 min with a high yield by using microwaves as energy source. When the reaction time was shortened to 30 s, conversion was reduced to 13%. As listed in Table 3, the use of different solvent results in different yields. Dimethyl sulfoxide (DMSO), a solvent in the high-ranking group with a large dielectric loss, showed a 100% conversion. Water, belonging to the middle group, showed a 28% conversion. Water has the highest dielectric constant, but its dielectric loss values ε and angles tan δ do not rank at the top of their respective list. Considering only the dielectric constant, one would assume that water is the most polar solvent in a microwave field. However, due to the comparatively low dielectric loss values and angles, water should be classified as a medium absorber material. Upon conventional heating, a mixture of THF, toluene, *t*-BuOH, and water gave a high yield due to specific hygroscopic properties [32]. Next, the reactivity in the H_2_O/*t*-BuOH solvent system was examined in a series of experiments in which the quantity of the catalyst was changed. When the concentration of the catalyst was reduced to 1.0 mol% and 0.5 mol%, a conversion of over 99% was obtained. At a catalyst concentration of 0.3 mol%, the conversion was reduced to 37%. However, using DMSO, which has a larger, the reaction proceeded with only 0.3 mol% of the catalyst. When the reaction time was reduced to 30 s, the reaction proceeded smoothly, such that the quantity of the catalyst could be reduced to 0.1 mol%. Interestingly, when the reaction was carried out without a solvent (entry 16), the yield was dramatically improved to 100%. Even when using a 0.1 mol% catalyst, a satisfactory conversion of 96% was achieved (Table 3).

**Table 3:** Optimization of click reaction catalyzed by CuO/AB.

Entry	Cat (mol%)	Time (min)	Solvent	Conv (%) ^a^
1	3	300	H_2_O/*t*-BuOH(2:1)	100 ^b^
2	3	60	H_2_O/*t*-BuOH(2:1)	91 ^c^
3	3	1	H_2_O/*t*-BuOH(2:1)	100
4	3	0.5	H_2_O/*t*-BuOH(2:1)	10
5	3	1	H_2_O	28
6	3	1	*t*-BuOH	100
7	3	1	DMSO	100
8	3	1	DMF	100
9	3	1	1-BuOH	100
10	3	1	2-BuOH	11
11	3	1	Toluene	0
12	3	1	THF	0
13	1	1	H_2_O/*t*-BuOH(2:1)	>99
14	0.5	1	H_2_O/*t*-BuOH(2:1)	>99
15	0.3	1	H_2_O/*t*-BuOH(2:1)	37
16	0.3	1	─	100
17	0.3	0.5	─	13
18	0.1	1	─	96
19	0.3	1	DMSO	100
20	0.3	0.5	DMSO	100
21	0.1	1	DMSO	94
22	─	1	─	3

*Reaction conditions*: benzyl azide (1 mmol) and phenylacetylene (1.5 mmol). ^a^ Determined by ^1^H-NMR. Yields are based on the amount of benzyl azide used; ^b^ Conventional thermal heating at 50 °C; ^c^ Conventional thermal heating at 100 °C.

Good results were attained upon using various terminal alkynes (Table 4). When the phenyl group was directly linked to the reactive azide, phenyl azide gave the expected 1,4-diphenyl-1*H*-1,2,3-triazole in the form of a single regioisomer with 100% conversion (entry 1, Table 4). Acetylenes, such as propynoic acid ethyl ester, reacted efficiently with the benzyl azide. The corresponding triazole, 1-benzyl-4-(phenoxymethyl)-1*H*-1,2,3-triazole, was relatively sluggish (entry 8 Table 4). The reaction with aliphatic alkynes, such as ethynyltrimethylsilane, gave high yields (entry 3, Table 4). Electron-donating or -withdrawing groups at the benzyl azides only slightly affected the reactivity [19]. *p*-Cl, *p*-methoxy, *o*-methoxy groups gave the expected 1-(4-chlorophenyl)-4-phenyl-1*H*-1,2,3-triazole, 1-(4-methoxyphenyl)-4-phenyl-1*H*-1,2,3-triazole, and 1-(2-methoxyphenyl)-4-phenyl-1*H*-1,2,3-triazole in the form of single regioisomers with 88%, 85%, and 75% conversions, respectively (entries 4, 5, and 6 in Table 4). Use of alkynes containing electron-withdrawing substituents, such as ethyl propiolate, significantly altered the reactivity (entry 8, Table 4). In some cases, electron-donating or -withdrawing groups on the benzyl azides greatly affected the reactivity. Electron-withdrawing groups impeded the reaction with corresponding lower yields. Among these groups, nitrogen dioxide in *para* position and OMe in *ortho* configuration exhibited the largest effect (entries 6 and 10, Table 4). Nevertheless, a single regioisomer was still produced, and yields of the products remained excellent.

## 6. Water-Soluble Block Copolymer Nanoreactors for the Synthesis of CuO Nanoparticles and Their Application in Click Chemistry

To date, a number of methods have been described to prepare Cu_2_O NPs with different particle size, morphology, and properties. The various preparation techniques include controlled thermal decomposition, chemical reduction with a proper surfactant, electrochemical reduction, microemulsion and the use of reverse micelles in a supercritical solvent. In all these methods, control of the particle size and morphology is achieved by the use of either a templating material or a capping reagent during NP growth. 

**Scheme 4:**
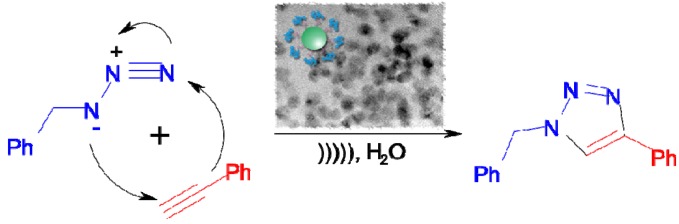
Ultrasound-assisted click reaction using water-soluble block copolymer nanoreactor.

Block copolymers have received much attention as attractive templates or scaffolds for engineering inorganic nanostructured materials, especially to control the size and spatial arrangement of NPs [38,39] (Scheme 4). Chemical activation by ultrasound, a nonconventional energy source, has become a very popular and useful technology in organic chemistry [40]. Several examples of ultrasound-assisted reactions have indicated high yields and short reaction times, and applications of this energy transfer process onto click reactions have been published [41]. However, performing click chemistry in aqueous media is still challenging due to the absence of a stable and active catalyst that is water-soluble [42]. Aqueous click chemistry has the economic, environmental, and processing benefits of both homogeneous aqueous catalysis and aqueous two phase catalysis. Water clearly stands out as the solvent of choice, with its fast reaction rate, high yield, selectivity, cheapness, “green” solvent nature, and environmental acceptability. 

**Table 4:** Results for solvent-free microwave promoted [3+2] cycloaddition of various azides with terminal alkynes in the presence of CuO/AB.

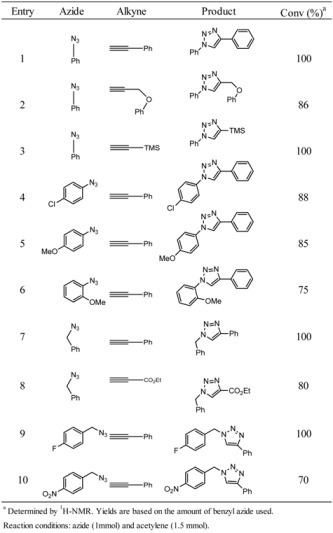

The original sizes and structures of the CuO NPs prepared by use of DHBC nanoreactors were preserved during catalytic transformations. The absolute amount of Cu that was used for click chemistry was determined by employing inductively coupled plasma optical-emission spectroscopy (ICP-OES). The CuO NPs within a DHBC shell showed excellent catalytic activity towards a wide range of azides and acetylenes.

Using water-soluble CuO nanoparticles (1 mol%), 1-benzyl-4-phenyl-1*H*-1,2,3-triazole was obtained with more than 99% conversion within 10 min (entry 4, Table 5). In general, it was found that increasing the reaction temperature and time were effective means of increasing the conversion (entries 2 and 3, Table 5). Using water-soluble CuO NPs (0.5 mol%) as the catalyst was used, a yield of 38% was achieved under the same conditions (entry 6, Table 5). Remarkably, the water-soluble CuO NPs were separated via centrifugation after the reaction, and they could be reused three times without any catalytic activity loss under identical reaction conditions. These results confirm that water-soluble CuO NPs represent an alternative catalytic system that satisfies the conditions for heterogeneous catalysis (easy separation, recyclability, and persistence).

**Table 5:** Optimization of the click reaction catalyzed by water-soluble CuO nanoparticle.

Entry	Cat (mol %)	Temp (°C)	Time (h)	Conv. (%) ^a^
1	1 mol% CuO-poly	25	3	3 ^b^
2	1 mol% CuO-poly	50	10 min	17
3	1 mol% CuO-poly	100	5 min	37
4	1 mol% CuO-poly	100	10 min	>99
5	1 mol% CuO-poly	100	10 min	27 ^c^
6	0.5 mol% CuO-poly	100	10 min	38
7	Recovered # 4	100	10 min	99
8	Recovered # 7	100	10 min	100

*Reaction conditions*: benzyl azide (0.80 mmol) and phenylacetylene (1.18 mmol) in H_2_O 4.5 mL. ^a^ Determined by ^1^H-NMR. Yields are based on the amount of benzyl azide used; ^b^ Transitional stirring; ^c^ Conventional thermal heating at 100 °C.

## 7. ZnO-CuO Core-Branch Nanocatalysts for Ultrasound-Assisted Click Reaction

Hybrid NPs offer multi-functionality with synergistic effects of independent domains [43,44]. From the viewpoint of heterogeneous catalysis, these hybrid NPs could supply optimal architectures for bifunctional catalytic systems [45,46,47]. Simply stated, one of the components serves as an active surface for the reaction, while the other behaves as a support to stabilize the entire structure. The inorganic interface between the two components can either tailor the chemical nature of the active component or generate new species to enhance the catalytic activity.

**Scheme 5:**
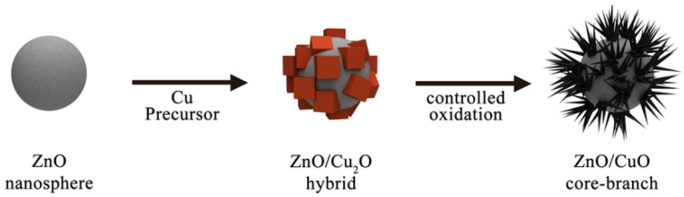
Synthetic scheme of ZnO–CuO core–branch hybrid nanoparticles.

In the present study, we combined the concepts of bifunctional catalysts and branched morphology in a colloidal metal-oxide system [27]. The ZnO–Cu_2_O hybrid NPs were synthesized by reduction of a copper precursor onto polycrystalline ZnO spheres. The resulting hybrid structures, so-called ZnO-CuO core-branch NPs, have active facets, and defects as well as large surface areas, and therefore, they are considered to be promising for catalytic applications. The ZnO-CuO core-branch NPs were synthesized via a two-step process from ZnO nanospheres (Scheme 5).

**Figure 3:**
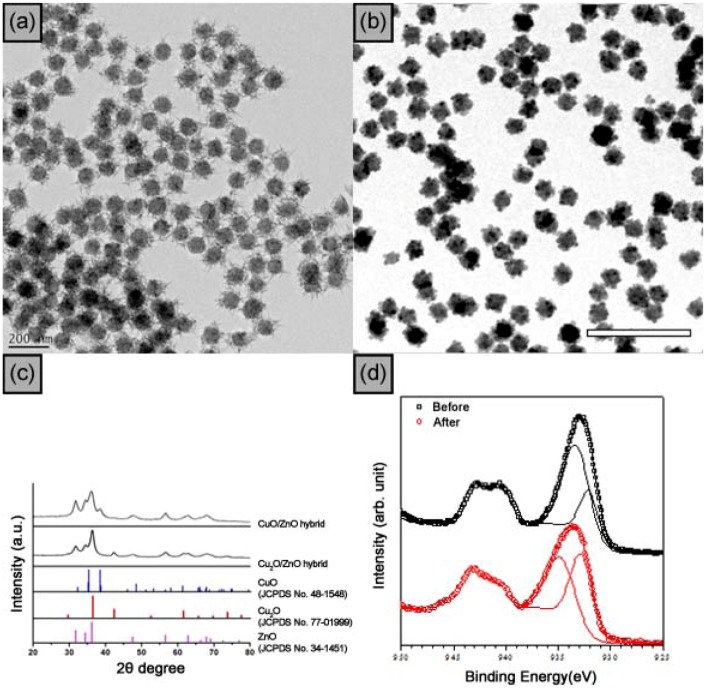
(**a**) ZnO/CuO core-branch nanoparticles; (**b**) TEM images of ZnO/Cu_2_O core-shell hybrid nanoparticles; (**c**) XRD spectra of ZnO/Cu_2_O and ZnO/CuO hybrid nanoparticles; (**d**) XSP spectra of ZnO/CuO hybrid nanoparticles. (Color online) The core-level XPS spectra of Cu 2p_3/2_ before and after chemical reaction. The solid curves represent the best-fitted results. The scale bars represent 200 nm (**a**,**b**).

The TEM image in Figure 3b shows that Cu_2_O nanocubes with an average edge size of 27 ± 4 nm were attached on the ZnO surface. The XRD pattern is a combination of wurtzite ZnO and primitive cubic Cu_2_O reflections (Figure 3c, JCPDS No. 77-0199). The distance between neighboring lattice fringes in the HRTEM image is 0.21 nm, in good agreement with the distance between {200} crystallographic planes of the Cu_2_O phase. The TEM image shows needle-like CuO branches with an average length of 49 ± 4 nm and a thickness of 8 nm (Figure 3a). Figure 3d shows the core-level x-ray photoelectron spectroscopy (XPS) spectra of Cu 2p_3/2_ of the CuO/ZnO NPs before and after the chemical reaction. The spectra were fitted after removing a Shirley background. The solid curves represent the best-fitting results. The XPS spectra can be deconvoluted into two peaks for both cases. The lower (higher) binding energy (BE) might be attributed to the Cu 2p_3/2_ state of Cu_2_O (CuO). After chemical reaction, the two peaks are shifted towards higher BE. However, the relative areal intensity ratio between Cu_2_O and CuO increased and reached the same, suggesting that the system changed into Cu_2_O after the chemical reaction. Any remaining CuO remaining might be due to the air-contamination during sample transport for the XPS measurements.

To demonstrate the prominence of the ZnO-CuO core-branch NPs inorganic catalysis, the particles were employed as a heterogeneous catalyst for cycloaddition reactions of benzyl azide and phenylacetylene to yield 1,4-disubstituted 1,2,3-triazoles. The catalytic reactions were carried out in a mixed solvent of H_2_O and *t*-BuOH (2:1) at room temperature. The ZnO-CuO nanocatalysts with a concentration of 3 mol% exhibited a conversion yield of 47%, under vigorous stirring for 3 h.

Notably, the catalytic performance was largely enhanced by applying ultrasound (Table 6). Ultrasound irradiation has been widely used in synthetic chemistry to induce mechanical effects on heterogeneous reactions [40]. Under the present reaction conditions with a ZnO-CuO nanocatalyst, under ultrasound irradiation, the yield increased to complete conversion within 10 min at room temperature (entry 2, Table 6). The reaction did not proceed without ultrasound. CuO hollow nanospheres, one of the best catalysts among Cu-based heterogeneous systems [17,19], activated the reaction with a yield of 14%, whereas commercially available mm-sized CuO particles proceeded the reaction with a yield of 1% under identical conditions (entries 5 and 6, Table 6). After the reaction, the ZnO–CuO NPs were readily recovered by centrifugation and were reused five times with only a slight loss of their catalytic activity (entries 8–12, Table 6). 

**Table 6:** [3+2] Azide–alkyne cycloaddition reactions catalyzed by the ZnO–CuO hybrid nanocatalysts under ultrasonic irradiation ^a^.

Entry	Cat (mol%)	Time (min)	Temp.	Conv. ^b^ (%)
1	ZnO-CuO (3 mol%)	5	R. T.	80
2	ZnO-CuO (3 mol%)	10	R. T.	100
3	ZnO-CuO (1 mol%)	10	R. T.	21
4	ZnO nanoparticles (3 mol%)	10	R. T.	N.R.
5	CuO hollows (3 mol%)	10	R. T.	14
6	Commercial CuO ^c^ (3 mol%)	10	R. T.	<1
7	ZnO-Cu_2_O (3 mol%)	10	R. T.	31
8	Recovered from #2	10	R. T.	100
9	Recovered from #8	10	R. T.	100
10	Recovered from #9	10	R. T.	100
11	Recovered from #10	10	R. T.	82
12	Recovered from #11	10	R. T.	76

^a^ *Reaction conditions*: benzyl azide (0.6 mmol) and phenylacetylene (0.9 mmol) in H_2_O-*t*-BuOH (2 : 1); ^b^ Determined by ^1^H-NMR spectra; ^c^ Commercial CuO purchased from Aldrich (cat. no. 544868).

## 8. Conclusions

We have synthesized various CuO NPs, specifically CuO hollow nanospheres, CuO hollow NPs on AB, DHBC nanoreactors for the synthesis of CuO nanoparticles and ZnO-CuO hybrid NPs, and we have used non-conventional energy sources like microwaves and ultrasound with the aim of achieving [3+2] cycloaddition of azides with terminal alkynes. The reaction products are predictably and reliably obtained, showing in all cases the exclusive formation of the 1,4-isomer.

CuO hollow NPs have shown a good catalytic activity. We have additionally loaded this catalyst onto AB to solve the recovery problem. The recycling of catalysts will give positive economical and environmental effect. Solvent-free microwave irradiation also exhibited good conversion yield in click reactions. Since organic synthesis in aqueous media is more challenging, we synthesized water-soluble DHBC nanoreactors for the synthesis of CuO NPs and performed ultrasound-assisted click reactions. The use of water as the solvent results in a high reaction rate, yield, and selectivity. Furthermore, it is cheap, and environmentally friendly. Synergistic effects between ZnO and CuO domains could be observed in ZnO-CuO hybrid NPs with a high activity and reusability. We expect to produce good catalytic systems for many reactions, including click reactions. 
